# Coincidental Loss of Bacterial Virulence in Multi-Enemy Microbial Communities

**DOI:** 10.1371/journal.pone.0111871

**Published:** 2014-11-03

**Authors:** Ji Zhang, Tarmo Ketola, Anni-Maria Örmälä-Odegrip, Johanna Mappes, Jouni Laakso

**Affiliations:** 1 Centre of Excellence in Biological Interactions, Department of Biological and Environmental Science, University of Jyväskylä, Jyväskylä, Finland; 2 Department of Biological and Environmental Science, University of Helsinki, Helsinki, Finland; Virginia Tech, United States of America

## Abstract

The coincidental virulence evolution hypothesis suggests that outside-host selection, such as predation, parasitism and resource competition can indirectly affect the virulence of environmentally-growing bacterial pathogens. While there are some examples of coincidental environmental selection for virulence, it is also possible that the resource acquisition and enemy defence is selecting against it. To test these ideas we conducted an evolutionary experiment by exposing the opportunistic pathogen bacterium *Serratia marcescens* to the particle-feeding ciliate *Tetrahymena thermophila*, the surface-feeding amoeba *Acanthamoeba castellanii*, and the lytic bacteriophage Semad11, in all possible combinations in a simulated pond water environment. After 8 weeks the virulence of the 384 evolved clones were quantified with fruit fly *Drosophila melanogaster* oral infection model, and several other life-history traits were measured. We found that in comparison to ancestor bacteria, evolutionary treatments reduced the virulence in most of the treatments, but this reduction was not clearly related to any changes in other life-history traits. This suggests that virulence traits do not evolve in close relation with these life-history traits, or that different traits might link to virulence in different selective environments, for example via resource allocation trade-offs.

## Introduction

Compared to the vast knowledge on the prevention and treatment of bacterial infectious disease, relatively little is known about how the virulence of bacteria has evolved. Virulence evolution is often exemplified as a tug of war between the multicellular host and the pathogen, where the virulence (the degree of host damage or mortality caused by the pathogen) [1] evolves solely through host-pathogen interaction [2]–[4]. Contrary to this idea, the “coincidental evolution of virulence hypothesis” suggests that virulence evolves indirectly due to selection forces that are not related to the host-pathogen interaction *per se*, but because of selection that occurs outside host environments [2], [3], [5], [6]. This is a plausible expectation when considering opportunistic, environmentally growing bacterial pathogens because they typically live in a complex web of interactions with biotic and abiotic selection pressures that might not be directly connected to their potential hosts [7].

In the natural environment, top-down regulation by bacteriophages and protozoans are two major biotic causes of bacterial mortality [8], [9]. In order to survive, bacteria have evolved wide arrays of defence mechanisms against their natural enemies [10], [11]. These adaptations have also been suggested to alter the virulence of the bacteria [11]–[13]. For example, a biofilm-forming ability can effectively lower predation pressure by ciliate predators that prey in the open water. However, the biofilm-forming ability of many bacteria can also be directly linked to the virulence of bacteria as it can prevent macrophage phagocytosis inside the multicellular host [11], [14]–[16].

In addition to the means that prevent predator ingestion in the first place, bacteria have evolved ways to survive the ingestion process and even benefit from it [11]. Survival and reproduction inside protozoan predators, especially in amoebae, may have even contributed to the evolution of several bacterial pathogens [11]. Therefore, virulence could have evolved via adaptations to survive inside protozoan food vacuoles, which could then promote survival within phagocytes in the immune system [17], [18]. Perhaps the most typical example of this type of evolution is *Legionella pneumophila* causing Legionnaires' disease. This species is sometimes found as a parasite of free-living amoeba [19]–[21]. However, infection of the human body is an evolutionary dead end for *L. pneumophila* because human-to-human transmission is unlikely [22], [23]. This suggests that the virulence traits of *L. pneumophila* are not evolved from human-bacteria interaction, but rather “coincidentally” via amoeba-bacteria interaction [2], [24]. In fact this linkage is assumed strong enough that the virulence of bacterial clones are frequently assayed indirectly via amoebae resistance tests [25]–[31].

Bacteriophages can also have a profound impact on the evolution of bacterial virulence. Bacteriophages are known to carry important virulence genes [32]–[34]. For example, they have been found to contain genes encoding exotoxins and other virulence factors that can be horizontally transferred into the bacterial genome [35], [36]. Moreover, bacteria can alter their cell surface antigens to evade phage adsorption [10], whilst host immune systems rely on bacterial surface antigens to identify bacterial invaders [37], [38]. Thus bacteriophage selected bacterial surface antigens could indirectly affect host entry, either positively or negatively.

Although protozoan predators and bacteriophages could potentially contribute to elevated bacterial virulence, outside-host defensive adaptations can also be costly and traded off with virulence related traits [39], [40]. For example, when bacteria experience protozoan predation, the motility of bacteria that is sometimes positively linked to virulence [41], [42] can trade off with anti-predator traits resulting in lowered virulence [39]. It has also been shown that elevated outside-host temperature can select for higher virulence in *Serratia marcescens*, while coevolution with phage can counteract this effect [43]. Moreover high virulence in *Salmonella typhimurium* can be costly in terms of reduced growth in the outside host environment because of the expression of virulence factor (type III secretion system) in a non-host environment [44]. The nutritional conditions of the bacterial growth environment can also significantly affect bacterial metabolism and the expression of virulence factors [45]–[47]. For example, it has been found that the virulence of the pathogenic fungi was negatively correlated to the carbon-to-nitrogen (C∶N) ratio of the culturing medium [48]–[50]. Therefore, if a similar correlation occurs for bacteria, then the costly virulence traits might be selected against during a prolonged period in a non-host environment. In conclusion, the environmental lifestyle can attenuate or strengthen the virulence depending on the selection forces in the system [7].

Although predators are supposed to play an important role in the evolution of virulence, experiments testing this theory are rare and the studies that do exist only consider a single predator system [40], [51], [52]. However, in a natural environment it is more conceivable that several predators are present simultaneously, potentially complicating the picture considerably. To test how virulence and other life-history traits evolve in complex enemy communities, we cultured the facultative pathogen *S. marcescens* either alone or with three types of common bacterial predators (amoeba, ciliate and bacteriophage in all seven possible combinations) in a simulated pond water environment for 8 weeks. *S. marcescens* is a gram-negative opportunistic pathogen infecting a broad spectrum of hosts, including plants, corals, nematodes, insects, fish and mammals [57], [58]. They can also be found free-living in soil, freshwater, and marine ecosystems [59], [60] making it likely that *S. marcescens* frequently encounters parasitic and predatory organisms. Notably, *S. marcescens* is also capable of re-entering the environment after decomposing the host. This creates the possibility that the pathogen virulence is selected in nature by both environmental and host-pathogen interactions. During the experiment we followed the population dynamics of the prey bacterium. Due to the presumed importance of predators on the evolution of the bacterial virulence [28], [53], [54], the amoeba densities were also followed throughout the experiment. After the evolution experiment, a library containing 384 differentially evolved clones was built to detect changes in virulence, growth ability, biofilm-forming ability and amoeba resistance. The virulence of the ancestor and the evolved bacteria, *S. marcescens* Db11 was quantified in the fruit fly (*Drosophila melanogaster*) hosts via an oral infection model [55]. Since phagocytes play a vital role in the clearance of the Db11 from the hemolymph in this animal model [55], we believed that choosing the bacterial strain and infection model was relevant to our study. We hypothesized that if *S. marcescens* Db11 gained amoeba-resistance in the presence of amoeba predation, this resistance could be used to fight against phagocytes in the hemolymph, and thus gain higher virulence. With data from a multi-predator experiment we can test if the bacterial virulence is selected for, a result that is expected in the presence of bacterial enemies (phage, ciliate and amoebae), especially amoebae. However, it is also plausible that selection pressures by bacterial enemies could select against virulence [39], [40].

## Methods

### Study species

*Serratia marcescens* Db11 [56], [57] was initially isolated from a dead fruit fly and was kindly provided by Prof. Hinrich Schulenburg. The predatory particle feeding ciliate, *Tetrahymena thermophila* (strain ATCC 30008) has a short generation time of ca. 2 h [61] and was obtained from American Type Culture Collection. It is routinely maintained in PPY (Proteose Peptone Yeast Medium) at 25°C [51], [62]. The free-living amoeba, *Acanthamoeba castellanii* (strain CCAP 1501/10) has a generation time ca. 7 h [62] and was obtained from Culture Collection of Algae and Protozoa (Freshwater Biological Association, The Ferry House, Ambleside, United Kingdom) and routinely maintained in PPG (Proteose Peptone Glucose Medium) [63] at 25°C. Obligatory lytic bacteriophage Semad11, capable of infecting *S. marcescens* Db11, was isolated from a sewage treatment plant in Jyväskylä, Finland in 2009. No specific permission was required for collection or location of the bacteriophage. Semad11 is a T7-like bacteriophage belonging to Podoviridae (A.-M. Örmälä-Odegrip, unpublished data).

The evolution experiment was performed in New Cereal Leaf - Page's Amoeba Saline Solution (NAS) medium which was prepared as follows: 1 g of cereal grass powder (Aldon Corp., Avon, NY) was boiled in 1 liter of dH_2_O for 5 minutes, and then filtered through a glass fiber filter (GF/C, Whatman). After cooling, 5 ml of both PAS stock solutions I and II were added before being made up to a final volume of 1 litre with deionized water [64], [65].

Before the experiment started, the organisms were cultured separately and prepared as follows: bacterial culture, a single colony of *S. marcescens* was seeded to 80 ml of NAS medium in a polycarbonate Erlenmeyer flask capped with a membrane filter (Corning). The flask was incubated at 25°C on a rotating shaker (120 rpm) for 48 hours. The amoeba and ciliate cells were harvested and washed twice in 40 ml of PAS (Page's Amoeba Saline) with centrifugation at 1200× g for 15 min to pellet the cells. After the centrifugation, cells were suspended in PAS and adjusted to a final concentration of ca. 10 cells µl^−1^. To prepare the bacteriophage stock, LB-Soft agar (0.7%) from semi-confluent plates was collected and mixed with LB (4 ml per plate), and incubated for 3.5 h at 37°C. Debris was removed by centrifugation for 20 min at 9682× g at 5°C. Stock was filtered with 0.2 µm Acrodisc Syringe Filters (Pall). The bacteriophage stock was diluted 1∶100,000 in NAS medium, giving approximately 10^6^ plaque-forming unit (PFU) ml^−1^.

### Evolution experiment

**Figure 1:**
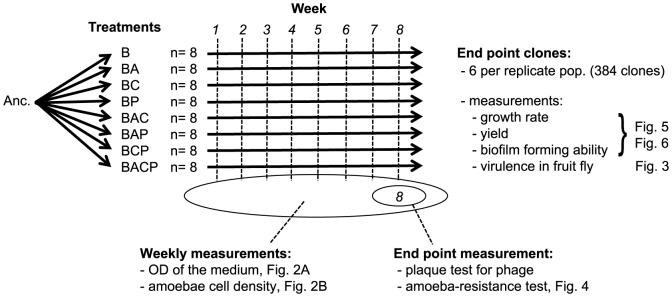
Schematic overview of our experimental evolution study, number of replicate populations, legends for treatments (Anc.: ancestral bacterial strain DB 11; B: bacteria; A: amoebae; C: ciliate; P: phage), and descriptions of different measurements.

The bacterium *S. marcescens* was either cultured alone or in a co-culture with the ciliates, amoebae and bacteriophages enemies 8 combinations (B, BA, BC, BP, BAC, BAP, BCP and BACP; B: bacteria; A: amoebae; C: ciliate; P: phage; Figure 1) for 8 weeks. Each treatment was replicated in 8 flasks. The experiment was initiated in 25 cm^2^ polystyrene flasks with 0.2 µm hydrophobic filter membrane caps (Sarstedt). Each flask was inoculated with 1 ml of the appropriate microorganism suspension and then the total volume was adjusted to 15 ml with NAS medium. The static liquid cultures were incubated at 25°C and 50% of the medium were replaced weekly with fresh NAS medium, making the system a pulsed resource type [66], [67]. Static liquid culture would create a spatial structuration that was similar to the pond water environment. All samples were taken just before the weekly medium renewal (Figure 1).

### Measurements during the evolution experiment

#### Bacterial biomass dynamics

Bacterial biomass in the free water phase was measured from 5 separate 200 µl samples from each flask on 100-well Honeycomb plates (Oy Growth Curves Ab Ltd). The amount of biomass was measured as optical density (OD) at 460–580 nm wavelength using Bioscreen C spectrophotometer (Oy Growth Curves Ab Ltd). The measurements were repeated 10 times at 5 min intervals. The mean of the measurements was used in the data analysis. To measure the amount of *S. marcescens* biofilm attached to the flask walls after 8 weeks had expired, 15 ml of 1% crystal violet solution (Sigma-Aldrich) was injected to the flasks. After 10 minutes, the flasks were rinsed 3 times with distilled water, and then 15 ml of 96% ethanol was added to flasks to dissolve crystal violet from the walls for 24 hours [68]. The amount of biofilm was quantified with the OD of the crystal violet-ethanol solution at 460–580 nm with Bioscreen C spectrophotometer [66].

#### Amoeba population dynamics

To follow the population dynamics of the amoeba, we measured the density of amoeba cells attached on the flask well. This measurement largely reflects the amoeba population dynamics in the flasks since the proportion of floating cells and cysts would be minimal after 7 days culture in the static cultures. In brief the flasks were carefully flipped and images (total area 5.23 mm^2^) of the flask wall were digitized with an Olympus SZX microscope (32× magnification). The amoeba cells attached to the flask wall were counted with a script developed in our lab for the Image Pro Plus software (v. 7.0) (Material S1). To determine the ciliate density by the end of the experiment, 250 µl of open water sample was mixed with 10 µl Lugol solution and injected into a glass cuvette rack (depth 2.34 mm). For each sample, 8 randomly placed images (total area 41.84 mm^2^) were digitized with an Olympus SZX microscope (32× magnification). The cell numbers in each image were counted with an Image Pro Plus script [69].

#### Detecting phage presence

To detect if the bacteriophages were present in the microcosms throughout the experiment and to detect possible contamination, we took 3 independent 500 µl samples from all flasks at the end of evolution experiment. The samples were treated with chloroform and centrifuged to remove bacteria, amoebas and ciliates. 10 µl supernatant drops were then added to 1.5% agar plates. The upper layer of the each plate was covered with 0.7% LB-agar that was mixed with 200 µl of overnight grown *S. marcescens* Db11 ancestor cells. The plates were incubated overnight in 25°C and the presence of phage plaques were checked.

### Measurements after the evolution experiment

#### Amoeba plaque test

After the evolution experiment was finished, half of the flasks from each treatment were randomly sampled to test for any resistance of the bacteria to amoeba predation. The test was adapted from a Wildschutte *et al.* [70] briefly, the flasks from the evolution experiments were shaken vigorously before 1 ml of the culture was transferred to a new tube containing 7 ml of dH_2_O. The tubes were mixed thoroughly and then centrifuged at 250 g for 10 min to bring down the suspended protozoan cells. 1 ml of the supernatant was spread evenly on to LN agar plates (PAS with 0.2% peptone, 0.2% glucose and 1% agar). A total of 10^5^ predatory amoeba cells (washed twice in PAS) suspended in 15 µl PAS solution were added to a sterile paper disk, and then placed in the middle of the plate. All the plates were incubated at 25°C for 8 days and then photographed. The images of the plates were used to measure plaque sizes with Image Pro Plus software (v. 7.0). A large plaque size indicates a small amoeba predation resistance.

#### Growth and biofilm forming ability of the individual clones

After the evolution experiment was complete, liquid samples from each replicate population of all treatments were streaked on three Luria–Bertani (LB) agar plates. They were incubated for 48 hours at 25°C, two bacterial colonies were randomly picked from each plate and inoculated to 5 ml LB liquid medium. The clones were grown at 25°C overnight on a shaker (120 rpm). To make stock cultures of the clone library, 200 µl of the liquid culture of each strain was mixed with 200 µl of 80% glycerol on 100-well Honeycomb plates in a randomized order and stored at −80°C. Prior to clonal growth measurements stock cultures from the clone library were inoculated to 100-well Honeycomb plates, directly from the freezer, with a plate replicator (EnzyScreen). Each well of the 100-well Honeycomb plates contained 400 µl fresh LB liquid medium. The OD of each well was measured continuously without shaking for 30 hours in 5 min intervals to estimate the maximum growth rate and maximum population size of the clones. After 30 hours, 100 µl of 1% crystal violet solution (Sigma-Aldrich) was added to each well to quantify amount of biofilm that was produced. After 10 minutes incubation in 25°C, the plates were rinsed with distilled water 3 times and then 400 µl of 96% ethanol was added to each well and left for 24 hours to dissolve the crystal violet from the walls [68]. The amount of biofilm was quantified by measuring the OD of the crystal violet-ethanol solution at 460–580 nm with Bioscreen C spectrophotometer [66].

Identical measurements were also recorded with NAS medium either with or without the protozoan predators (amoeba or ciliate). The abovementioned clones were first grown in 400 µl LB liquid medium in 100-well Honeycomb plates. After incubation at 25°C for 24 h in static cultures, 10 µl of the bacterial culture was transferred to 100-well Honeycomb plates. Each well contained 390 µl NAS mixed with or without NAS washed amoeba (5–10 cells/µl) or ciliate cells (0.5–1 cells/µl). Subsequent measurements for growth and biofilm assay were performed as described above.

Estimation of growth rate and yield was based on the Matlab script written by TK that fits linear regression to 25 time-points along a sliding data window with background correction, using ln-transformed OD data. The maximal growth rate is determined by finding the largest slope of linear regression within all fitted regressions for the particular clone. The yield was determined as the highest average OD over the 25 data point window.

#### Virulence of the evolved clones

Stock cultures of the clone library were inoculated to 100-well Honeycomb plates, filled with 400 µl fresh LB liquid medium with plate replicator (EnzyScreen). For a positive control the ancestor *S. marcescens* Db11 was added to two separate wells in each plate and used in the subsequent infection experiment. After 24 h incubation at 25°C without shaking, 800 µl of the bacterial culture was mixed with the 800 µl of 100 mM sucrose solution. The mixture was absorbed to cotton dental roll (Top Dent, Lifco Dental, Enköping, Sweden) folded on the bottom of a standard 75×23 mm fly vial (Sarstedt, Nümbrecht, Germany). 1600 µl of 100 mM sucrose solution was used as a negative control. Ten *D. melanogaster* adults (2–3 days old) from a large laboratory colony (Oregon R, kindly provided by Christina Nokkala from the University of Turku) were transferred to each vial and plugged with cotton. This was done for all the bacterial clones. Deaths of flies were monitored over next 4 days at 3–6 h intervals.

### Statistical analysis

Changes in bacterial density and amoeba density were compared using repeated measurements ANOVA. The effects of the evolutionary treatments on bacterial virulence were quantified with Cox regression by fitting evolutionary treatment and identity of population as categorical covariates. The amoeba plaque test and the amount of biofilm at the end of the evolution experiment were compared using ANOVA. All the analyses were done with SPSS v. 19 (IBM).

Life history and defensive traits of evolved clones were tested with ANOVA including treatment (all possible combinations of predators) as a fixed factor and population identity as a random factor. From the data we tested effects of treatments on growth rate, yield and biofilm, as well as growth rate and yield under the influence of ciliate or amoebae presence. Coevolution of traits was studied with MANOVA and subsequent eigenanalysis to reveal if changes in certain traits would lead to corresponding changes in other traits across the treatments. Thus, this analysis allows pinpointing strongly interconnected traits [71]. MANOVA and eigenanalysis was performed with MATLAB function manova1 (R2012a, Mathworks; Statistics toolbox) for population averaged trait values. Values used for MANOVA for virulence were hazard function coefficients averaged over the populations within each treatment.

Two replicated populations (one in the treatment BC and one in BAC) were found contaminated by Semad11 phages. Moreover, in two replicates of the treatment BACP phages were not detected by plaque assay. All the other samples from phage containing treatments formed phage plaques on the ancestor Db11 bacterial lawn. This confirms that phages did not go extinct during the experiment. The aforementioned 4 flasks were excluded from the data analysis.

## Results

### The population dynamics of bacterial prey and amoeba predators during the experiment

**Figure 2:**
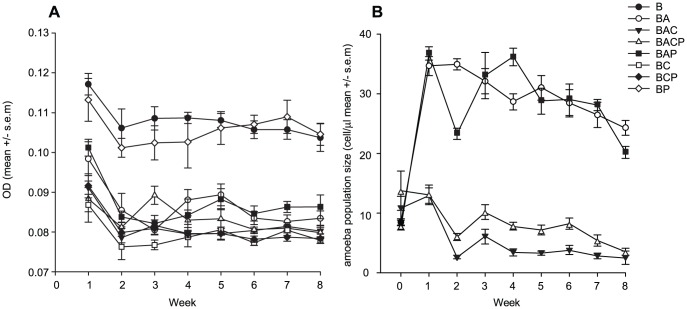
Bacterial biomass dynamics (A) and amoebae population dynamics (B) during the eight-week evolution experiment. The bacteria were reared alone or in several combinations of bacterial enemies (Anc.: ancestral bacterial strain DB 11; B: bacteria; A: amoebae; C: ciliate; P: phage). See Table S1 for pairwise comparisons.

The presence of predators generally reduced the bacterial biomass in free water phase (OD of the medium: F_7, 52_ = 674.620, p<0.001; Figure 2A). The ciliates reduced the biomass most dramatically: on average by 27% during the weeks 1–8 when compared to the control (B). Biomass reduction by ciliate and phage (BCP), and amoeba and ciliate (BAC) communities was 25%. Amoeba (BA) and amoeba and phage (BAP) communities reduced bacteria biomasses by 20%. The bacteriophage (BP) reduced the biomass only by 2%. The pairwise-comparisons of the rest of the treatments were significant after Bonferroni correction, except BCP vs. BC, and BACP vs. BAC.

The amount of biofilm produced in each treatment was different at week 8 when measured directly from the microcosm walls (ANOVA, F_7, 52_ = 39.101, p<0.001). The highest amount of biofilm was found in the presence of ciliates (BC) and the lowest amount of biofilm was found in the treatments BAC and BACP. Detailed pairwise comparison can be found in Table S1.

The amoeba population sizes declined in all treatments after the initial increase during the first week (F_3, 28_ = 280.257, p<0.001; Figure 2B). The amoeba population sizes were higher in amoeba (BA) and amoeba and phage (BAP) treatments (on average 30 cells µl^−1^) throughout the 8-week evolution experiment. Adding phage to the amoeba treatment did not change the population dynamics of the amoeba (Fisher's LSD: BA vs. BAP, p = 0.485). However, ciliates reduced the amoeba population sizes: on average only 5 cells µl^−1^ were found throughout the experiment in treatment BAC, and on average 8 cells/µl in treatment BACP.

### Virulence

**Figure 3:**
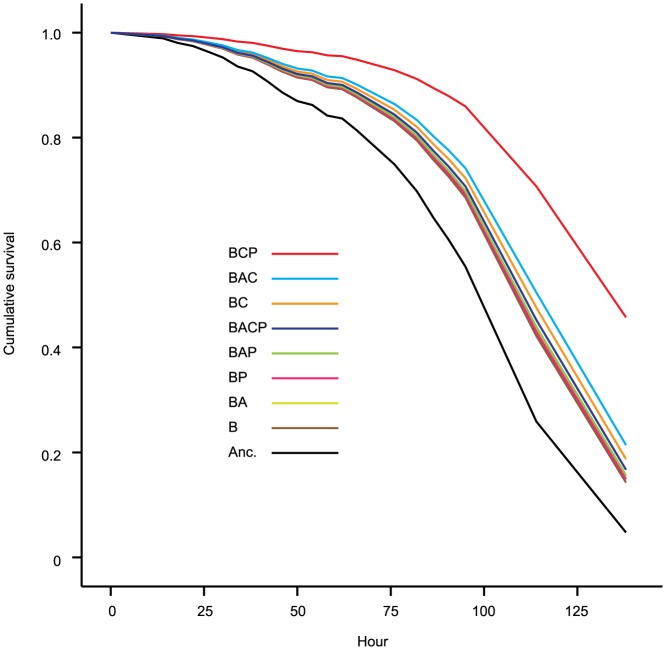
Cumulative survival curves of the fruit flies that were infected with evolved and ancestral bacterial clones. Anc.: ancestral bacterial strain DB 11; B: bacteria; A: amoebae; C: ciliate; P: phage. The survival curves represented the pooled survival data of the 480 fly individuals for each treatment (10 flies per vial, 6 clones per population and 8 replicates per treatment). The treatment codes are in the order of the increasing virulence. See Table S2 for pairwise comparisons.

In order to explore if past selection with predators had influenced virulence we utilized the *Drosophila* oral infection assay. The treatment group that had evolved with ciliates and phages (BCP) had clearly lower virulence than the rest of the evolved treatment groups (p<0.01 in all pairwise comparisons; Figure 3; Table S2). In this group the between population variation was also very high and statistically significant. In the majority of the other groups, the between population variation was clearly non-significant (p<0.135, but in BCP, p<0.001; BAC p = 0.003). Moreover, all treatments had lower virulence than the ancestor (p<0.01 in all pairwise comparisons; Figure 3; Table S2). There was no statistical support for the difference in virulence between any other treatment groups.

### Sensitivity to amoebae predation

**Figure 4:**
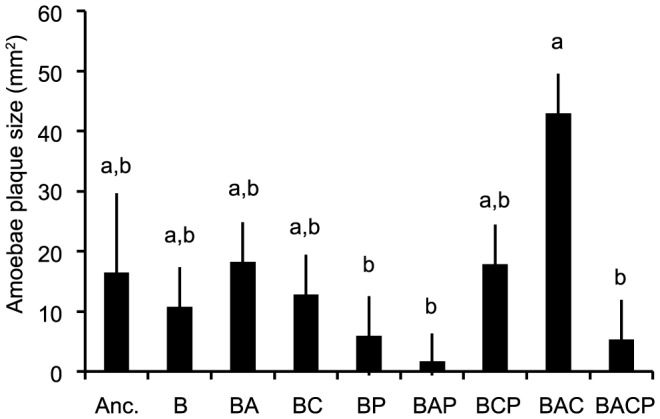
Sensitivity of evolved bacteria on amoebae predation measured using amoeba plaque test. Anc.: ancestral bacterial strain DB 11; B: bacteria; A: amoebae; C: ciliate; P: phage. Sensitivity is measured as a plaque size (mm^2^) in bacterial lawn caused by the introduced amoeba in semi-solid agar plate. Letters indicate if treatment means are statistically similar (p>0.05), after Bonferroni correction for multiple comparisons. Tests are based on the post hoc comparisons of estimated marginal means for treatments ANOVA. All bars correspond to 4 randomly picked samples from 8 replicate populations.

The amoeba plaque test revealed that the sensitivity to amoeba predation (measured with the area of visible plaque formed on bacterial lawn) was highest in bacteria co-cultured with amoeba and ciliates (BAC: p<0.05 in all pairwise comparisons; Figure 4; Table S1). The lowest amoeba sensitivity was found in the treatment where the amoeba were reared with phages (BAP; Figure 4) or with ciliates and phages (BACP; BAP vs. BACP: p = 0.66, Figure 4). The detailed result of pairwise comparisons can be found in the Table S1. Although the treatment group that had evolved with ciliates and phages (BCP) had the lower virulence than the other evolved treatment groups, its sensitivity to amoeba predation did not differ from the others in pairwise comparisons (Table S1), which suggests no clear link between amoebae predation and virulence.

### Life-history traits

**Figure 5:**
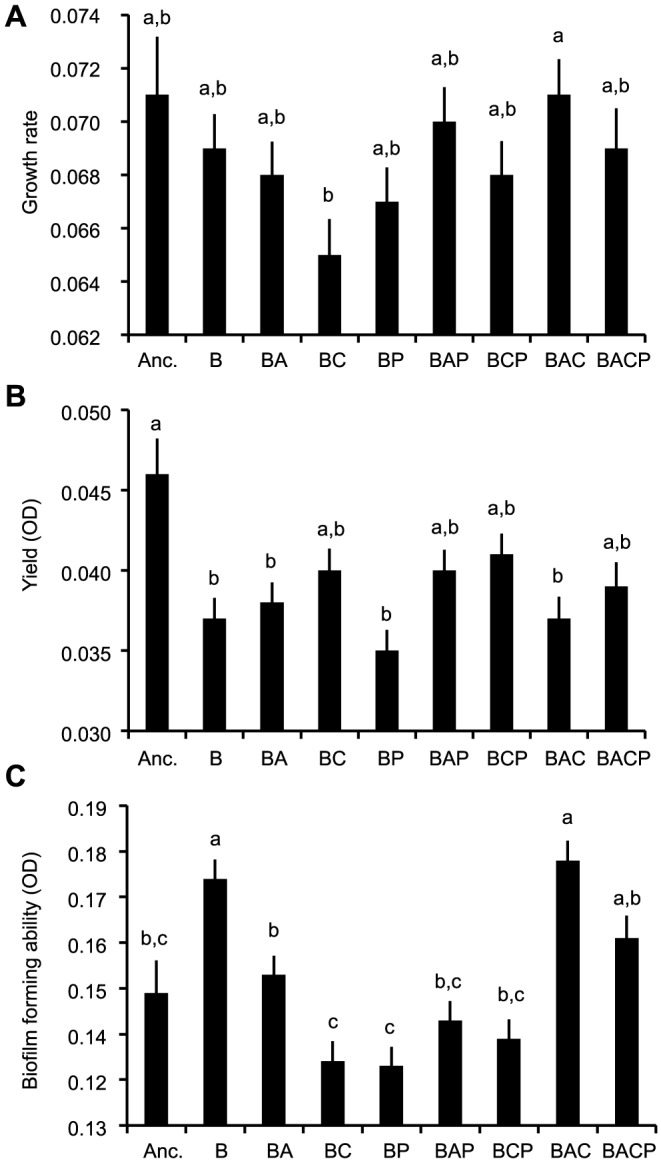
Growth rate (panel A), yield (panel B) and biofilm forming ability (panel C) differences between ancestral clones and clones that have evolved alone (B) or in different combinations of bacterial enemies. Anc.: ancestral bacterial strain DB 11; B: bacteria; A: amoebae; C: ciliate; P: phage. Letters indicate if treatment means are considered statistically similar (p>0.05) after Bonferroni correction for multiple comparisons. Tests are based on the post hoc comparisons of estimated marginal means for treatments of ANOVA testing the effects of treatment and population identity on these traits. Bars correspond to measurements of 6 clones from 8 replicate populations, in ancestor n = 16).

Treatments did not influence the maximal growth rate strongly (Table 1), however low growth rates were found in clones that had evolved with ciliates (BC; Figure 5A; Table S2). The ancestor clones had the largest yield. Evolutionary treatments did not differ greatly from each other but the clones that had evolved with phages had the lowest yield (BP; Figure 5B; Table S2). Evolutionary changes occurred most dramatically in the biofilm forming ability. The highest biofilm forming abilities were found from the clones that had evolved alone (B) or with amoeba and ciliate (BAC). Intermediate biofilm production was found in ancestral clones (Anc.) or if clones had evolved with amoebae (BA) or with all enemies (BACP). The lowest biofilm production was found if clones that had evolved with ciliate (BC), phage (BP), amoebae and phage (BAP) or with ciliate and phage (BCP) (Figure 5C; Table S1).

**Table 1:** Estimated (ANOVA) evolutionary effects of different combinations of enemies (treatment), and population identity on bacterial virulence against *Drosophila melanogaster*, and on bacterial life-history traits, measured alone or with amoebae or ciliate.

	Treatment			Population		
	Wald	df	p	Wald	df	p
Virulence	48.6	8	<0.001	169.58	52	<0.001
	F	df1,2	p	F	df	p
Growth rate	2.465	8,32.007	0.033	0.799	52,306	0.836
Yield	2.582	8,39.627	0.023	1.349	52,306	0.066
Biofilm	12.987	8,37.001	<0.001	1.097	52,306	0.311
	F	df1,2	p	F	df	p
Growth with amoebae	9.565	8,40.485	<0.001	1.456	52,306	0.029
Yield with amoebae	3.595	8,46.032	0.003	2.885	52,306	<0.001
Growth with ciliate	1.296	8,39.223	0.274	1.304	52,306	0.091
Yield with ciliate	4.757	8,40.432	<0.001	1.449	52,306	0.031

Wald denotes Wald's test statistics, and F corresponds to F-test statistics, df denote degrees of freedom and p indicate statistical significance.

**Figure 6:**
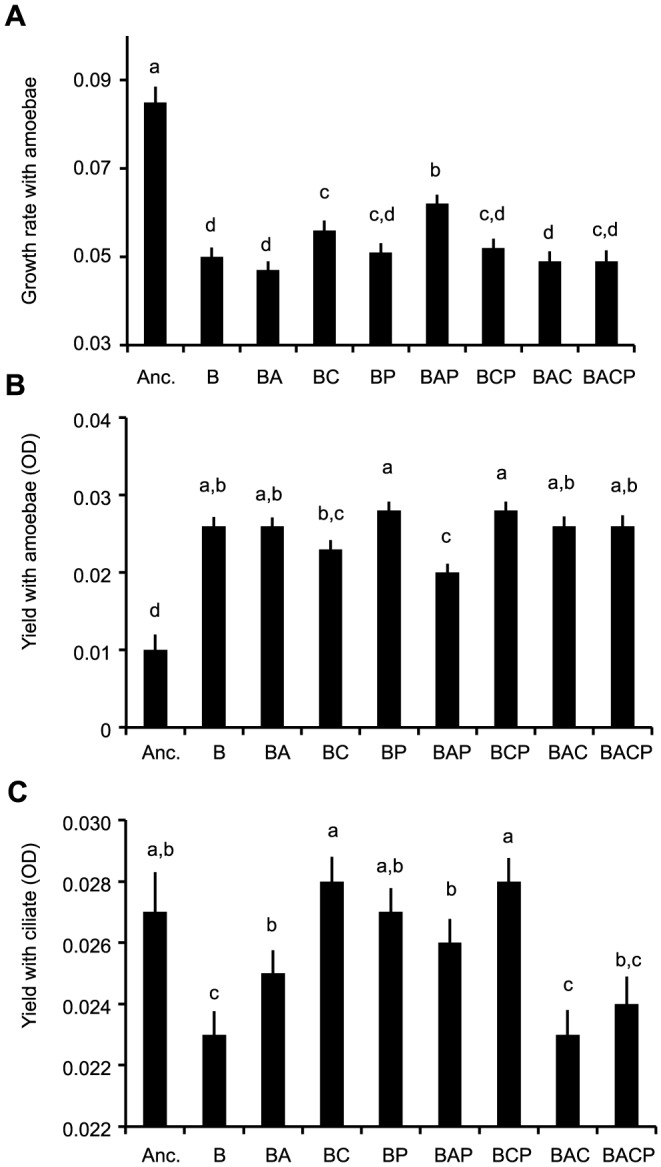
Growth rate with amoebae (panel A), yield with amoebae (panel B) and yield with ciliate (panel C) differences between ancestral clones and clones that have evolved alone or in different combinations of bacterial enemies. Anc.: ancestral bacterial strain DB 11; B: bacteria; A: amoebae; C: ciliate; P: phage. Letters indicate if treatment means are considered statistically similar after Bonferroni correction for multiple comparisons. Tests are based on the post hoc comparisons of estimated marginal means for treatments of ANOVA testing the effects of treatment and population identity on these traits. Bars correspond to measurements of 6 clones from 8 replicate populations, in ancestor n = 16).

From the defensive traits the strongest changes were observed in growth rate and yield when the bacteria were co-cultured with amoebae. In both of the traits ancestor bacteria deviated from the clones that had undergone evolutionary treatments; ancestors had higher growth rate co-cultured with amoebae but lower yield than evolved clones (Figure 6; Table S2). Similarly, from the evolved groups the highest growth rate was found from the group that had evolved with phage and amoebae (BAP), whereas its yield with amoebae was lowest (Figure 6; Table S2). Growth rate measurements did not indicate that treatments affected the resistance of clones against ciliate predators. However, yield with ciliates was lowest if bacteria had evolved alone (B) or with amoebae and ciliate (BAC), and highest if clones had evolved with ciliate (BC) and ciliate and phage (BCP) (Figure 6C; Table 1; Table S2).

### Coevolved traits

Based on the individual traits in different treatment combinations, it is difficult to get an idea of how co-ordinate traits evolved. Therefore we analysed all traits in multivariate ANOVA, followed by eigen-analysis. In this analysis we found that two dimensions dictated multivariate evolution amongst evolutionary treatments (support for two dimensions p = 0.028, for 1 dimension p<0.001). The first dimension was characterized by variation in biofilm and ciliate defence. Those treatments that exerted strong positive selection on biofilm production had a lower yield with ciliate in free water. The second dimension of trait evolution was formed by those treatments that had increased growth rate without predators and also had a lower growth rate with ciliate. Neither of the two “major” eigen-functions linked virulence to other traits (Table 2).

**Table 2:** Eigenvectors describing dimensions of multivariate evolution under different kind of enemies (loadings, i.e. correlations, of original variables to new composite variable, based on MANOVA.

	Eig. 1.	Eig. 2.	Eig. 3.	Eig. 4.	Eig. 5.	Eig. 6.	Eig. 7.	Eig. 8.	Eig. 9.
Biofilm	**0.753**	0.326	−0.205	0.215	0.229	0.376	−0.343	−0.024	0.183
Yield	0.131	0.257	−0.268	0.671	−0.023	−0.155	0.150	−0.199	−0.149
Yield with amoeba	0.081	−0.223	−0.276	−0.200	0.330	0.094	0.452	−0.459	0.678
Yield with ciliate	**−0.579**	−0.253	−0.032	0.472	−0.203	0.111	−0.665	0.115	−0.026
Growth rate	0.018	**−0.518**	0.498	0.241	−0.664	−0.375	−0.106	0.245	0.022
Growth with amoeba	−0.150	0.228	0.489	−0.143	0.321	0.501	0.335	−0.357	0.511
Growth with ciliate	0.222	**0.610**	−0.483	−0.356	0.075	−0.209	0.184	0.319	0.460
Virulence	0.037	−0.149	0.080	0.171	0.176	0.236	0.202	0.666	0.063
Eigenvalues	4.692	0.9220	0.5689	0.1967	0.1039	0.0394	0.0173	<0.001	<0.001
% explained	71.74	14.10	8.70	3.01	1.59	0.60	0.27	<0.001	<0.001

Below the eigenvectors are the eigenvalues i.e. amount of variation explained by eigenvectors and the percentage of the total variation explained. First eigen function (Eig1) describes contribution of individual traits to the largest difference between the treatments. In eigenvectors that are considered significant (Eig 1 and 2, see results) the largest contributors to the evolutionary differences are highlighted with bold.

When similar analysis was performed with information from the amoeba plaque test, a similar result was found, again supporting what was found for two multivariate dimensions (support for two dimensions p = 0.023, for 1 dimension p<0.001). The amoeba plaque test contributed moderately to a second eigenvector. This eigenvector had a slightly different composition than the analysis without the amoeba plaque test as the ltter contained all microcosm replicates. If anything, a higher resistance against amoebae was associated with higher biofilm forming ability and higher growth rate with ciliate. However, the amoeba plaque test clearly did not predict virulence. Since the amoeba plaque test was performed for a subset of the populations, their inclusion in more detailed measurements resulted in a smaller dataset. We base our discussion on the analysis of the larger and thus more reliable eigen-analysis without the amoeba plaque test (Table 2).

## Discussion

Predators such as ciliates and amebae, and parasitic phages are expected to be the main determinants of bacterial mortality in the natural environment [8], [9]. In addition to selection exerted by these predators and parasites on defensive traits, selections by bacterial natural enemies have often been suggested to lead to increased bacterial virulence [2], [5], [72] to such a degree that amoebae-resistance has been used as a direct proxy of strains' virulence [25], [27]–[31], [73]. However, we did not find evidence to suggest such a relationship as most of the experimental treatments had attenuated virulence. Moreover, there was no indication of coevolution of other life-history traits with virulence in evolved strains.

Contradictory to the theory that protozoan predators and phage parasites, amoebae in particular, play a strong role in the evolution of high virulence [11]–[13], [53], [72], [74], we found that virulence attenuated in all of the evolved populations regardless of the presence of the enemies. This is in accordance with the previous study with *S. marcescens* that suggest that ciliates can select for attenuated virulence [39], [40]. However, here we show that this is also the case with amoeba and phages [43], and under selection by multiple enemies at the same time. By far the strongest decrease in virulence was found in clones that had evolved with phages and ciliates. However, the between population variation in this group was very high and statistically significant. This was shown in separate analyses designed to test the amount of between population variance within treatments. In most of the other groups the between population variation was clearly non-significant (p<0.135, but in BCP, p<0.001; BAC p = 0.003). The between population variation is often seen as a signature of drift, mutation accumulation and lack of directional selection on traits [40], [75]–[78]. Thus, we propose that the strong decline of virulence in ciliate-phage treatment was primarily caused by the decay of unused traits through random mutation accumulation [40], [76].

To find out if evolutionary changes in virulence could be linked to changes in traits that are important for fitness outside the host, we measured growth parameters and resistance against amoeba and ciliates. However, none of the traits seemed to be determining the level of virulence amongst evolved strains. Thus, it is clear that resistance to protozoan predators or changes in other measured life-history traits are poor indicators for virulence in *S. marcescens*. However, in other bacterial species, virulence correlated positively with bacterial defences against predators [5], [12], [17], [26], [72] (but see [39], [40], [77]). In addition, several lines of research have emphasized the role of growth rate with virulence [79], [80] (but see [44], [77]). However, it could be that virulence might be traded off with whatever trait that is under selection in the given environment. This could have led to the clear lack of a connection between virulence and life history traits in an experiment where selection pressures are different between each treatment. This is a plausible outcome if virulence is traded off with life-history traits via finite resources. Then, any energetically costly trait under strong selection in outside-host environments could lead to virulence attenuation. Although we did not find a strong connection with virulence traits and life-history traits, we found that regardless of the evolutionary treatments, high biofilm-forming ability was closely linked with a low yield in a condition of co-culturing with ciliates in free water, and high growth rate was linked with low growth rate with ciliates. The first eigenvector (biofilm vs. yield with ciliates) could indicate that growth in biofilms does not lead to good protection against predation in free water, or that exposure to predation leads to biofilm formation only when predators are present and thus reduces cells in a free water environment. However, these findings from the eigen analysis effectively mean that there are evolutionary constraints between different life-history traits that remain unchangeable regardless of the evolutionary treatments. Moreover, since the predators (amoeba and ciliates) were effectively reducing the population densities in the long run (Figure 2A) the lack of selection on life history traits is not a plausible explanation for the obtained results.

When we compared ancestor's life history traits to evolved strains, it seemed that ancestors grew better with amoebae and were also the most virulent clone (Figure 3). However, another amoebae-resistance measurement, yield with amoebae, was actually lower for the ancestor strain than evolved strains. Moreover, in the amoebae-plaque test the ancestor strain did not excel in comparison to evolved strains. These contrasting results from measurements that should indicate the ability to resist amoebae suggest that there is a weak indication that amoebae-resistance evolved simultaneously with virulence. Therefore we suggest that the culture conditions could attenuate virulence, without any clear changes in life history traits. Interestingly, several experimental evolution studies have previously found that bacterial virulence decreases due to the exposure to the outside-host environment [39], [40], [81].

Alternatively, it has been suggested that if traits are not needed under particular conditions then their alleles become harmful and accumulate which leads to unused trait decay [76]. *S. marcescens* strain Db11 was isolated from a dead *Drosophila* fly over thirty years ago and has been routinely grown in highly protein-rich LB medium. Yet, the virulence of the strain has been maintained from lab to lab [55], [57], [82]. It is possible that the protein-enriched culture condition like LB medium (LB containing 10 g/l tryptone and 5 g/l yeast extract) was somehow needed for *S. marcescens* Db11 to maintain its virulence. However, a more likely scenario could be that our experimental conditions (low concentration of high C∶N ratio plant detritus) selected against virulence in the 8 week evolution experiment. In addition, there might be other unknown factors that could of affected the virulence. Although predators in general effectively lowered population sizes of the bacteria (Figure 2A), spatial heterogeneity and potentially other niches created by the static cultures might lower the strength of selection on defensive traits. For example, the biofilm could act as a protection against protozoan predation, and some bacteria might have not been under selection at all.

To summarize, we found no support for the idea that enemies outside the host could select for higher virulence, as all experimental treatments led towards lower bacterial virulence. Among evolved strains virulence was not linked to other life-history characters, suggesting that selective pressures from protozoan predators (ciliates and amoebae) and parasitic phages did not dictate virulence evolution. In conclusion, our dataset offered a case against coincidental evolution of the virulence hypothesis that expects outside-host selections, especially amoebae predation, would lead to higher bacterial virulence [2], [3], [5], [72].

## Supporting Information

Table S1 **Pairwise comparisons of experimental treatment differences on amoeba population sizes, biomass in the free water phase and attached biofilm at the end of the experiment.** Significant pairwise comparisons after Bonferroni correction are high-lighted with bold (critical α: 0.00178 = 0.05÷28, but amoebae population size critical α: 0.008 (0.05/6)). (B = bacteria alone, BC = with ciliate; BA with amoebae; BP with phage etc.. Anc. stands for ancestor Db11 strain).
(DOCX)

Table S2 **Pairwise comparisons of experimental treatment differences on measured virulence, growth and defensive traits.** Significant pairwise comparisons after Bonferroni correction are highlighted with bold (critical α: 0.00138 (0.05/36)) (B = bacteria alone, BC = with ciliate; BA with amoebae; BP with phage etc.. Anc. stands for ancestor Db11 strain).
(DOCX)

Material S1 **The macro for the Image-Pro Plus program to automatically count protozoan cells in microscopic images.**
(IPM)
