# Supplementary material for: Coincidental Loss of Bacterial Virulence in Multi-Enemy Microbial Communities
Source: PLoS One. 2014 Nov 3;9(11):e111871. doi: 10.1371/journal.pone.0111871 (PMC4218854; doi:10.1371/journal.pone.0111871)
Supplement: Table S1 — Pairwise comparisons of experimental treatment differences on amoeba population sizes, biomass in the free water phase and attached biofilm at the end of the experiment. Significant pairwise comparisons after Bonferroni correction are high-lighted with bold (critical α: 0.00178 = 0.05÷28, but amoebae population size critical α: 0.008 (0.05/6)). (B = bacteria alone, BC = with ciliate; BA with amoebae; BP with phage etc.. Anc. stands for ancestor Db11 strain). (DOCX) [file pone.0111871.s001.docx]

**Table S1. Pairwise comparisons of experimental treatment differences on amoeba population sizes, biomass in the free water phase and attached biofilm at the end of the experiment.** Significant pairwise comparisons after Bonferroni correction are high-lighted with bold (critical α: 0.00178=0.05 ÷ 28, but amoebae population size critical α: 0.008 (0.05/6)). (B=bacteria alone, BC=with ciliate; BA with amoebae; BP with phage etc.. Anc. stands for ancestor Db11 strain)

|  | *Biomass in free water* | | |  | | *Biofilm at week8* | | |  | | *Amoeba population size* | | |  |
| --- | --- | --- | --- | --- | --- | --- | --- | --- | --- | --- | --- | --- | --- | --- |
|  | | t | p | |  | | t | p | |  | | t | p | |
| B vs. BA | | 35.625 | **p<0.001** | |  | | 1 | 0.319 | |  | | NA | NA | |
| B vs. BC | | 45.671 | **p<0.001** | |  | | 7.667 | **p<0.001** | |  | | NA | NA | |
| B vs. BP | | 3.864 | **p<0.001** | |  | | 4.875 | **p<0.001** | |  | | NA | NA | |
| B vs. BAP | | 34.505 | **p<0.001** | |  | | 2.375 | 0.03 | |  | | NA | NA | |
| B vs. BCP | | 44.794 | **p<0.001** | |  | | 4.625 | **p<0.001** | |  | | NA | NA | |
| B vs. BAC | | 42.112 | **p<0.001** | |  | | 4.444 | **p<0.001** | |  | | NA | NA | |
| B vs. BACP | | 37.727 | **p<0.001** | |  | | 4.222 | **p<0.001** | |  | | NA | NA | |
| BA vs. BC | | 11.25 | **p<0.001** | |  | | 8.667 | **p<0.001** | |  | | NA | NA | |
| BA vs. BP | | 31.761 | **p<0.001** | |  | | 6 | **p<0.001** | |  | | NA | NA | |
| BA vs. BAP | | 1.12 | 0.268 | |  | | 1.25 | 0.225 | |  | | 0.707 | 0.485 | |
| BA vs. BCP | | 9.169 | **p<0.001** | |  | | 5.625 | **p<0.001** | |  | | NA | NA | |
| BA vs. BAC | | 7.691 | **p<0.001** | |  | | 3.444 | **0.001** | |  | | 22.225 | **p<0.001** | |
| BA vs. BACP | | 4.738 | **p<0.001** | |  | | 3.222 | 0.002 | |  | | 19.261 | **p<0.001** | |
| BC vs. BP | | 41.937 | **p<0.001** | |  | | 3.333 | **0.001** | |  | | NA | NA | |
| BC vs. BAP | | 12.334 | **p<0.001** | |  | | 9.778 | **p<0.001** | |  | | NA | NA | |
| BC vs. BCP | | 2.393 | 0.02 | |  | | 3.667 | **p<0.001** | |  | | NA | NA | |
| BC vs. BAC | | 3.448 | **0.001** | |  | | 12.111 | **p<0.001** | |  | | NA | NA | |
| BC vs. BACP | | 5.866 | **p<0.001** | |  | | 11.889 | **p<0.001** | |  | | NA | NA | |
| BP vs. BAP | | 30.64 | **p<0.001** | |  | | 7.25 | **p<0.001** | |  | | NA | NA | |
| BP vs. BCP | | 40.928 | **p<0.001** | |  | | 0.375 | 0.743 | |  | | NA | NA | |
| BP vs. BAC | | 38.378 | **p<0.001** | |  | | 8.778 | **p<0.001** | |  | | NA | NA | |
| BP vs. BACP | | 34.147 | **p<0.001** | |  | | 8.556 | **p<0.001** | |  | | NA | NA | |
| BAP vs. BCP | | 10.289 | **p<0.001** | |  | | 6.875 | **p<0.001** | |  | | NA | NA | |
| BAP vs. BAC | | 8.773 | **p<0.001** | |  | | 2.333 | 0.021 | |  | | 21.518 | **p<0.001** | |
| BAP vs. BACP | | 5.777 | **p<0.001** | |  | | 2.111 | 0.041 | |  | | 18.554 | **p<0.001** | |
| BCP vs. BAC | | 1.168 | 0.248 | |  | | 8.444 | **p<0.001** | |  | | NA | NA | |
| BCP vs. BACP | | 3.75 | **p<0.001** | |  | | 8.222 | **p<0.001** | |  | | NA | NA | |
| BAC vs. BACP | | 2.554 | 0.014 | |  | | 0.222 | 0.858 | |  | | 2.964 | **0.006** | |
