# Supplementary material for: Coincidental Loss of Bacterial Virulence in Multi-Enemy Microbial Communities
Source: PLoS One. 2014 Nov 3;9(11):e111871. doi: 10.1371/journal.pone.0111871 (PMC4218854; doi:10.1371/journal.pone.0111871)
Supplement: Table S2 — Pairwise comparisons of experimental treatment differences on measured virulence, growth and defensive traits. Significant pairwise comparisons after Bonferroni correction are highlighted with bold (critical α: 0.00138 (0.05/36)) (B = bacteria alone, BC = with ciliate; BA with amoebae; BP with phage etc.. Anc. stands for ancestor Db11 strain). (DOCX) [file pone.0111871.s002.docx]

**Table S2.** **Pairwise comparisons of experimental treatment differences on measured virulence, growth and defensive traits.** Significant pairwise comparisons after Bonferroni correction are highlighted with bold (critical α: 0.00138 (0.05/36)) (B=bacteria alone, BC=with ciliate; BA with amoebae; BP with phage etc.. Anc. stands for ancestor Db11 strain)

|  |  |  | *Virulence* | |  | *Amoeba resistance* | | *Growth rate* | | *Yield* |  | *Biofilm production* | | | *Growth with A* | | *Yield with A* | | *Growth with C* | | *Yield with C* | |
| --- | --- | --- | --- | --- | --- | --- | --- | --- | --- | --- | --- | --- | --- | --- | --- | --- | --- | --- | --- | --- | --- | --- |
|  |  |  | Wald | p |  | t | p | t | p | t | p | t | p |  | t | p | t | p | t | p | t | p |
| ANC | vs. | B | 7.337 | 0.007 |  | 0.387 | 0.702 | 0.577 | 0.565 | 3.648 | **p<0.001** | 3.136 | 0.002 |  | 8.512 | **p<0.001** | 7.011 | **p<0.001** | 0.741 | 0.459 | 2.590 | 0.010 |
| ANC | vs. | BA | 8.312 | 0.004 |  | 0.120 | 0.905 | 0.914 | 0.361 | 3.392 | **0.001** | 0.575 | 0.566 |  | 9.134 | **p<0.001** | 7.295 | **p<0.001** | 0.352 | 0.725 | 0.735 | 0.463 |
| ANC | vs. | BC | 12.106 | **0.001** |  | 0.248 | 0.806 | 2.309 | 0.022 | 2.403 | 0.017 | 1.773 | 0.077 |  | 6.950 | **p<0.001** | 5.737 | **p<0.001** | 0.731 | 0.465 | 1.247 | 0.213 |
| ANC | vs. | BP | 7.596 | 0.006 |  | 0.714 | 0.481 | 1.362 | 0.174 | 4.496 | **p<0.001** | 1.913 | 0.057 |  | 8.225 | **p<0.001** | 7.849 | **p<0.001** | 0.455 | 0.650 | 0.648 | 0.517 |
| ANC | vs. | BAP | 8.590 | 0.003 |  | 1.055 | 0.300 | 0.468 | 0.640 | 2.381 | 0.018 | 0.698 | 0.486 |  | 5.282 | **p<0.001** | 4.433 | **p<0.001** | 1.134 | 0.258 | 0.101 | 0.920 |
| ANC | vs. | BCP | 35.036 | **p<0.001** |  | 0.095 | 0.925 | 1.114 | 0.266 | 2.333 | 0.020 | 1.178 | 0.240 |  | 8.028 | **p<0.001** | 7.984 | **p<0.001** | 0.526 | 0.599 | 1.237 | 0.217 |
| ANC | vs. | BAC | 13.509 | **p<0.001** |  | 1.796 | 0.083 | 0.182 | 0.856 | 3.708 | **p<0.001** | 3.483 | **0.001** |  | 8.532 | **p<0.001** | 6.927 | **p<0.001** | 0.935 | 0.350 | 2.258 | 0.025 |
| ANC | vs. | BACP | 10.063 | 0.002 |  | 0.758 | 0.455 | 0.576 | 0.565 | 2.673 | 0.008 | 1.412 | 0.159 |  | 8.255 | **p<0.001** | 6.704 | **p<0.001** | 0.206 | 0.837 | 1.511 | 0.132 |
| B | vs. | BA | 0.809 | 0.368 |  | 0.801 | 0.430 | 0.468 | 0.640 | 0.387 | 0.699 | 3.606 | **p<0.001** |  | 0.806 | 0.421 | 0.339 | 0.735 | 1.535 | 0.126 | 2.619 | 0.009 |
| B | vs. | BC | 2.046 | 0.153 |  | 0.220 | 0.828 | 2.394 | 0.017 | 1.651 | 0.100 | 6.705 | **p<0.001** |  | 2.009 | 0.045 | 1.638 | 0.102 | 2.014 | 0.045 | 5.243 | **p<0.001** |
| B | vs. | BP | 0.652 | 0.419 |  | 0.518 | 0.609 | 1.093 | 0.275 | 1.183 | 0.238 | 7.027 | **p<0.001** |  | 0.401 | 0.689 | 1.162 | 0.246 | 0.399 | 0.690 | 4.509 | **p<0.001** |
| B | vs. | BAP | 0.883 | 0.347 |  | 1.121 | 0.272 | 0.152 | 0.880 | 1.763 | 0.079 | 5.337 | **p<0.001** |  | 4.497 | **p<0.001** | 3.589 | **p<0.001** | 2.611 | 0.009 | 3.467 | **0.001** |
| B | vs. | BCP | 17.088 | **p<0.001** |  | 0.761 | 0.453 | 0.748 | 0.455 | 1.850 | 0.065 | 6.032 | **p<0.001** |  | 0.709 | 0.479 | 1.328 | 0.185 | 0.303 | 0.762 | 5.349 | **p<0.001** |
| B | vs. | BAC | 3.024 | 0.082 |  | 3.452 | 0.002 | 1.035 | 0.302 | 0.146 | 0.884 | 0.531 | 0.596 |  | 0.168 | 0.867 | 0.001 | 1.000 | 0.280 | 0.780 | 0.415 | 0.678 |
| B | vs. | BACP | 1.271 | 0.260 |  | 0.586 | 0.562 | 0.032 | 0.974 | 1.097 | 0.274 | 2.132 | 0.034 |  | 0.141 | 0.888 | 0.014 | 0.989 | 1.228 | 0.221 | 1.301 | 0.194 |
| BA | vs. | BC | 0.282 | 0.596 |  | 0.582 | 0.565 | 1.961 | 0.051 | 1.290 | 0.198 | 3.251 | **0.001** |  | 2.816 | 0.005 | 1.984 | 0.048 | 0.535 | 0.593 | 2.739 | 0.007 |
| BA | vs. | BP | 0.007 | 0.932 |  | 1.319 | 0.198 | 0.637 | 0.525 | 1.583 | 0.115 | 3.495 | **0.001** |  | 1.211 | 0.227 | 0.836 | 0.404 | 1.132 | 0.259 | 1.941 | 0.053 |
| BA | vs. | BAP | 0.002 | 0.969 |  | 2.047 | 0.050 | 0.621 | 0.535 | 1.394 | 0.164 | 1.787 | 0.075 |  | 5.350 | **p<0.001** | 3.965 | **p<0.001** | 1.103 | 0.271 | 0.887 | 0.376 |
| BA | vs. | BCP | 11.412 | **0.001** |  | 0.040 | 0.968 | 0.285 | 0.776 | 1.480 | 0.140 | 2.470 | 0.014 |  | 1.527 | 0.128 | 1.002 | 0.317 | 1.237 | 0.217 | 2.783 | 0.006 |
| BA | vs. | BAC | 0.777 | 0.378 |  | 2.650 | 0.013 | 1.502 | 0.134 | 0.526 | 0.599 | 4.058 | **p<0.001** |  | 0.617 | 0.538 | 0.330 | 0.742 | 1.781 | 0.076 | 2.139 | 0.033 |
| BA | vs. | BACP | 0.048 | 0.827 |  | 1.388 | 0.176 | 0.398 | 0.691 | 0.750 | 0.454 | 1.168 | 0.244 |  | 0.599 | 0.549 | 0.326 | 0.745 | 0.174 | 0.862 | 1.096 | 0.274 |
| BC | vs. | BP | 0.375 | 0.540 |  | 0.737 | 0.467 | 1.326 | 0.186 | 2.807 | 0.005 | 0.161 | 0.872 |  | 1.617 | 0.107 | 2.771 | 0.006 | 1.624 | 0.105 | 0.835 | 0.405 |
| BC | vs. | BAP | 0.242 | 0.623 |  | 1.375 | 0.180 | 2.542 | 0.012 | 0.071 | 0.943 | 1.490 | 0.137 |  | 2.385 | 0.018 | 1.870 | 0.062 | 0.536 | 0.592 | 1.853 | 0.065 |
| BC | vs. | BCP | 8.580 | 0.003 |  | 0.542 | 0.592 | 1.676 | 0.095 | 0.147 | 0.883 | 0.847 | 0.398 |  | 1.326 | 0.186 | 2.946 | 0.003 | 1.728 | 0.085 | 0.039 | 0.969 |
| BC | vs. | BAC | 0.140 | 0.708 |  | 3.232 | 0.003 | 3.354 | **0.001** | 1.758 | 0.080 | 7.076 | **p<0.001** |  | 2.129 | 0.034 | 1.603 | 0.110 | 2.243 | 0.026 | 4.723 | **p<0.001** |
| BC | vs. | BACP | 0.101 | 0.751 |  | 0.806 | 0.427 | 2.183 | 0.030 | 0.453 | 0.651 | 4.112 | **p<0.001** |  | 1.996 | 0.047 | 1.501 | 0.134 | 0.510 | 0.660 | 3.576 | **p<0.001** |
| BP | vs. | BAP | 0.015 | 0.901 |  | 0.524 | 0.605 | 1.245 | 0.214 | 2.944 | 0.003 | 1.690 | 0.092 |  | 4.097 | **p<0.001** | 4.755 | **p<0.001** | 2.212 | 0.028 | 1.042 | 0.298 |
| BP | vs. | BCP | 11.799 | **0.001** |  | 1.279 | 0.211 | 0.351 | 0.725 | 3.037 | 0.003 | 1.033 | 0.303 |  | 0.307 | 0.759 | 0.160 | 0.873 | 0.098 | 0.922 | 0.819 | 0.413 |
| BP | vs. | BAC | 0.916 | 0.338 |  | 3.969 | **p<0.001** | 2.103 | 0.036 | 1.009 | 0.314 | 7.396 | **p<0.001** |  | 0.560 | 0.576 | 1.134 | 0.257 | 0.669 | 0.504 | 3.991 | **p<0.001** |
| BP | vs. | BACP | 0.092 | 0.762 |  | 0.069 | 0.946 | 0.976 | 0.330 | 2.188 | 0.029 | 4.346 | **p<0.001** |  | 0.510 | 0.610 | 1.085 | 0.279 | 0.860 | 0.391 | 2.857 | 0.005 |
| BAP | vs. | BCP | 11.412 | **0.001** |  | 2.001 | 0.055 | 0.900 | 0.369 | 0.077 | 0.938 | 0.667 | 0.506 |  | 3.812 | **p<0.001** | 4.939 | **p<0.001** | 2.322 | 0.021 | 1.867 | 0.063 |
| BAP | vs. | BAC | 0.777 | 0.378 |  | 5.107 | **p<0.001** | 0.887 | 0.376 | 1.869 | 0.063 | 5.745 | **p<0.001** |  | 4.562 | **p<0.001** | 3.507 | **0.001** | 2.830 | 0.005 | 2.973 | 0.003 |
| BAP | vs. | BACP | 0.048 | 0.827 |  | 0.444 | 0.660 | 0.172 | 0.864 | 0.529 | 0.597 | 2.788 | 0.006 |  | 4.287 | **p<0.001** | 3.298 | **0.001** | 1.179 | 0.239 | 1.896 | 0.059 |
| BCP | vs. | BAC | 6.361 | 0.012 |  | 2.690 | 0.012 | 1.770 | 0.078 | 1.954 | 0.052 | 6.425 | **p<0.001** |  | 0.862 | 0.389 | 1.297 | 0.196 | 0.577 | 0.564 | 4.808 | **p<0.001** |
| BCP | vs. | BACP | 10.339 | **0.001** |  | 1.348 | 0.189 | 0.656 | 0.512 | 0.602 | 0.547 | 3.415 | **0.001** |  | 0.795 | 0.427 | 1.238 | 0.217 | 0.954 | 0.341 | 3.621 | **p<0.001** |
| BAC | vs. | BACP | 0.463 | 0.496 |  | 4.038 | **p<0.001** | 0.989 | 0.324 | 1.210 | 0.227 | 2.581 | 0.010 |  | 0.017 | 0.986 | 0.988 | 0.015 | 1.462 | 0.145 | 0.892 | 0.373 |
